# Autonomic Nervous System Response Patterns of Test-Anxious Individuals to Evaluative Stress

**DOI:** 10.3389/fpsyg.2022.824406

**Published:** 2022-02-28

**Authors:** Wenjun Bian, Xiaocong Zhang, Yunying Dong

**Affiliations:** ^1^Department of Psychology, School of Medicine and Holistic Integrated Medicine, Nanjing University of Chinese Medicine, Nanjing, China; ^2^School of Education, Jiangsu University of Technology, Changzhou, China

**Keywords:** test anxiety, HRV, sympathetic nerve, parasympathetic nerve, evaluative stress

## Abstract

Test anxiety is a widespread and primarily detrimental emotion in learning and achievement settings. This research aimed to explore the autonomic nervous system (ANS) response patterns of test-anxious individuals in response to evaluative stress. By presenting a standard interview task, an evaluative scenario was effectively induced. Heart rate variability (HRV), a biomarker that can accurately reflect the ANS activity, was used to reflect the physiological responses of 48 high test-anxious subjects and 49 low test-anxious subjects. Results indicate that: (1) both groups show a significantly increased emotional arousal in the evaluative scenario; (2) high test-anxious individuals show a significantly decreased emotional pleasantness in the evaluative scenario, whereas low test-anxious individuals show no significant changes; (3) both groups show a significantly increased low-frequency HRV; (4) high test-anxious individuals show a significantly decreased high-frequency HRV and root mean square of successive heartbeat interval differences (RMSSD), whereas low test-anxious individuals remain stable. These findings suggest that high test-anxious individuals display an increased sympathetic nervous system (SNS) activity and a decreased parasympathetic nervous system (PNS) activity in response to evaluative stress, while low-anxious individuals display an increased SNS activity and a stable PNS activity in response to evaluative stress.

## Introduction

Test anxiety refers to an individual’s disposition to respond to excessive worry, intrusive thoughts, mental disorganization, tension, and physiological arousal when exposed to evaluative situations (Zeidner, 2014). In China, more than 20% of college students, middle school students, and primary school students generally experience high test anxiety (Huang and Zhou, 2019). Students are frequently confronted with evaluative scenarios, including exams, interviews, public presentations, and athletic competitions. These scenarios that include evaluative elements (i.e., teacher invigilation, scoring by judges, classmate comparison, and video surveillance) easily induce students’ stress responses, as their performance in these scenarios may affect the opportunities for scholarships, advancement, or employment opportunities. Frequent exposure to evaluative stress is detrimental to students’ mental and physical health (Loft et al., 2007).

Test anxiety can amplify individuals’ stress responses in an evaluative scenario. High test-anxious (HTA) individuals are evaluated for larger cardiovascular stress responses (i.e., increased heart rate, higher systolic and diastolic blood pressure) than low test-anxious (LTA) individuals (Roos et al., 2021). Studies show that LTA and HTA individuals react differently to physiological arousal signals. LTA individuals typically regard arousal as a positive signal. They are more likely to mobilize additional cognitive resources to handle challenges and put more effort into the current scenario. HTA individuals tend to regard the arousal as a negative signal. They consider the scenario a threat and try to escape to avoid it (Hollandsworth et al., 1979; Zhang et al., 2015). Avoiding a threat is an instrumental defense behavior to protect individuals from life-threatening consequences, thus ensuring adaptation to changing environmental conditions (Hamm and Weike, 2005; Boeke et al., 2017). If avoidance behaviors become too dominant, they may impair psychosocial function (Krypotos, 2015). Alternatively, avoidance behavior toward threats occupies attentional resources and causes fewer resources for other cognitive processes (Zhang et al., 2018; Wei et al., 2021). These results shed light on the understanding of why HTA individuals typically fail to perform well in exams.

To achieve a more coherent picture of the physiological arousal of test-anxious individuals, researchers are trying to explore the neural mechanism of physiological arousal from the perspective of the autonomic nervous system (ANS). The two branches of the ANS, the sympathetic nervous system (SNS) and the parasympathetic nervous system (PNS), are a coordinated response system underlying physiological arousal, stress, and flight/fight behavior (Porges, 1992). The SNS would be activated by evaluative scenarios. This activation is evolutionarily regarded as the tuning of the SNS to ensure metabolic resources to defend against attacks (Fanselow, 1994; Lang et al., 1997). Although SNS activation is an adaptive response to threats, high levels of SNS activation would have detrimental effects in other aspects. The SNS activation induced by an evaluative scenario makes individuals fatigue and interferes with their performance (Yoshie et al., 2009). The response of the PNS is related to inhibitory control, attentional regulation, and emotion regulation. The Poly-Vagal theory suggests that the myelinated vagus actively inhibits SNS activation, enabling regulatory behaviors (e.g., self-soothing and inhibiting arousal) that facilitate a calm behavioral state (Porges, 2007, 2009).

Furthermore, the pairing of the SNS and PNS plays an important role in the stress responses of test-anxious individuals, serving as an important neural mechanism of test anxiety. The ANS has different activation patterns: there are nine pairing patterns of sympathetic and parasympathetic nerves (Yan et al., 2006). Specifically, in addition to mutually antagonistic patterns, there are other pairing patterns: simultaneous enhancement, simultaneous weakening, or one strengthening and the other unchanged. Up to now, far too little attention has been paid to the ANS response patterns of test-anxious individuals. Previous studies on test anxiety typically employ heart rate (HR) and blood pressure (BP) as measures to assess physiological arousal (Roos et al., 2021). These measures are innervated by both the SNS and the PNS, so researchers cannot clarify whether physiological arousal is produced by increased SNS activity or decreased PNS activity.

Due to the variety of ANS response patterns, researchers need to select more sensitive measures to distinguish between SNS and PNS activity. HRV, a non-invasive and convenient biomarker, is reliable for studying the mechanism of the SNS and PNS responses (Heiss et al., 2021). Low-frequency (LF) HRV (0.04–0.15 Hz) and high-frequency (HF) HRV (0.15–0.40 Hz) are the most commonly used frequency-domain measures. It is generally accepted that LF mainly reflects SNS activity, while HF reflects PNS activity (Holzman and Bridgett, 2017). Root mean square of successive heartbeat interval differences (RMSSD), a commonly used time-domain measure of heart period variability, can reflect PNS activity (Yoshie et al., 2009).

The study about the ANS response patterns of test-anxious individuals can provide new insights into treatments for alleviating test anxiety. Previous studies have shown that biofeedback training, relaxation training, and meditation effectively alleviate excessive autonomic arousal of test-anxious individuals (Huntley et al., 2019). Nevertheless, substantial differences in the ANS mechanism exist among treatments targeting test anxiety. According to research by Wells et al. (2012), heart rate variability biofeedback (HRVB) reduces anxiety in a stressful scenario mainly by enhancing PNS activity. Sutarto et al. (2013) found that HRVB improves cognitive performance by enhancing SNS activity. Based on the controversy about the mechanism of these treatments, the ANS response patterns of test-anxious individuals in response to evaluative stress should be clarified, consequently setting training goals and evaluating training effects more accurately.

Putwain et al. (2021) suggest that future studies should have broader research scenarios to explore the neural mechanism of test anxiety. The interview is one of the most widely used testing methods in large-scale examinations in China (e.g., the national entrance examination for postgraduate and national civil servant examination). During a demanding interview, interviewers can assess interviewees’ diverse capabilities (e.g., professional knowledge, on-the-spot adaptability, and psychological quality). Studies on test anxiety usually set an evaluative scenario through subject examinations, IQ tests, and cognitive tests (Lang and Lang, 2010; Ramirez and Beilock, 2011; Zhang et al., 2015). Therefore, we set a standard stress interview task to create an evaluative scenario that included common stressors: social evaluation (which comes from scoring by judges, classmate comparison, and video surveillance) and monetary incentives (which reward those with high scores). The experiment aimed to explore the ANS response patterns of test-anxious individuals in response to evaluative stress and further to shed light on clinical psychological treatments for test anxiety.

## Materials and Methods

### Participants

Four hundred and twenty college students from different majors who participated in a public course of mental health completed the Test Anxiety Inventory (TAI). Students with the top 20% and bottom 20% TAI scores were invited to participate in the experiment. Finally, 48 HTA college students (*mean* age = 19.65 ± 1.18 years; *mean* TAI score = 52.02 ± 7.70) and 49 LTA college students (*mean* age = 19.90 ± 1.05 years; *mean* TAI score = 27.96 ± 3.31) completed the experiment. We found no significant difference in the ages of both groups (*t* = −1.12, *p* > 0.05, Cohen’*d* = −0.22) and found a significant difference in the TAI scores of both groups (*t* = 19.93, *p* < 0.001, Cohen’*d* = 4.06). Each participant signed an informed consent form and was compensated for their participation. This study was approved by the Ethics Committee of Nanjing University of Chinese Medicine.

### Measures

#### Test Anxiety Inventory

The TAI is a widely used self-report instrument that has been found to be valid and reliable (Spielberger, 1980). Participants rated the frequency of specific anxiety symptoms they experienced before, during, and after exams on a four-point scale. Studies have shown that the Chinese version of the TAI has high reliability and validity among undergraduate students (Wang, 2003). The Cronbach’s alpha coefficient for this measure is 0.90. We used the TAI before our experiment to assess the test anxiety level of students and selected the students whose scores were in the top 20% (representing HTA) and the bottom 20% (representing LTA) to participate in our experiment.

#### Emotional Experience Report

To assess the subjective emotional experience during the experiment, we instructed the participants to score their emotional pleasantness (1 refers to extremely unpleasant and 9 refers to extremely pleasant) and emotional arousal (1 refers to extremely calm and 9 refers to extremely nervous) on a nine-point scale after each experimental phase (Wei, 2020).

#### Manipulation of Evaluative Stress

We set an evaluative scenario with a standard interview task, following the methodology of Lü et al. (2018). The experiment presented the instrument via a computer screen (the computer played a recorded audio instruction simultaneously): “You will participate in a job interview for a primary or secondary teacher.” You have 30 s to prepare and 5 min to explain why you are qualified for the position. Your performance will be videotaped and evaluated by two interviewers. The interviewee with the highest score will receive ¥200 cash. “After the instrument, two interviewers entered the experimental room and sat in front of the subject, turning on the camera simultaneously.” After 30 s of preparation, the participants would deliver their job presentation for 5 min. If they paused for more than 10 s, they were reminded to continue. If participants were unable to continue, interviewers would ask standard questions (i.e., “Do you have experience in this job?” or “What are your plans if you get the job?”).

### Experimental Design

We used a 2 × 3 mixed experimental design, with test anxiety (HTA and LTA) as a between-subject variable, experimental phases (baseline phase, stress phase, and recovery phase) as a within-subject variable, HRV values (Ln LF, Ln HF, and Ln RMSSD) and emotional experience (pleasantness and arousal) as dependent variables.

### Procedures

**FIGURE 1:**
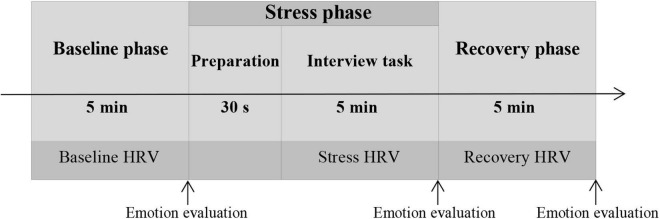
Experimental flow diagram.

When participants arrived at the laboratory, they signed an informed consent form. Following that, they naturally sat on a chair to relax, and researchers helped the subjects carry the sensor of a multiple-lead physiological recording instrument. Physiological data were collected during the procedure. After a 10-min rest, the experiment began. The experimental trial comprised the following three experimental phases (Figure 1).

#### Baseline Phase (5 Min)

Subjects sit peacefully on a chair, maintaining a stable breath, and gazing at a neutral picture (sourced from the International Affective Picture Library, IAPS) on the computer screen. At the end of this phase, the subjects scored their emotional pleasantness and emotional arousal.

#### Stress Phase (5 Min)

Subjects delivered a job interview presentation as directed (see section “Manipulation of Evaluative Stress”). At the end of this phase, the subjects scored their emotional pleasantness and emotional arousal once again.

#### Recovery Phase (5 Min)

Subjects were allowed to relax and look at the neutral picture on the computer screen again. After this phase, the subjects provided final assessments of their emotional pleasantness and emotional arousal.

### Physiological Data Acquisition

The ECG amplifier (ECG100C) of a Biopac MP150 multiple-lead physiological recorder was used to collect physiological data. Three Ag-AgCL disposable electrodes were pasted on the subjects’ wrist and ankles by the standard lead II configuration. The Biopac amplifier employed a band-pass filter of 35 Hz and 0.5 Hz, sampling at 1,000 Hz. Subsequently, AcqKnowledge 4.2 software was used to calculate the physiological scores of each participant during different phases. The data were subjected to a natural logarithmic transformation to obtain a normal distribution. The units of Ln LF and Ln HF are ms^2^. The unit of Ln RMSSD is ms.

### Statistical Analysis

All statistical tests were conducted with SPSS (Ver. 22, IBM Chicago, IL, United States). To assess the difference between HTA and LTA individuals during three experimental phases, we conducted a series of two-factor repeated measures ANOVAs, with test anxiety (HTA vs. LTA) as a between-subject variable and experimental phases (baseline phase, stress phase, and recovery phase) as a within-subject variable. These ANOVAs were conducted for subjective emotional experience (emotional pleasantness and arousal) and HRV values (Ln LF, Ln HF, and Ln RMSSD). When the interaction effect between the experimental phase and test anxiety was significant, a simple effect analysis would be conducted. Effect sizes were presented as partial η^2^ for ANOVA effects. All tests were two-tailed and were analyzed using a set level of significance of alpha = 0.05.

## Results

Table 1 shows the data of HRV and subjective emotional experience of HTA and LTA subjects in three experimental phases (baseline phase, stress phase, and recovery phase).

**TABLE 1:** Emotional experience and HRV values in three experimental phases (mean ± standard deviation).

Subjects	Experimental phase	Arousal	Pleasantness	Ln LF	Ln HF	Ln RMSSD
LTA (*n* = 49)	Baseline phase	2.96 ± 1.34	5.55 ± 1.26	6.42 ± 0.83	6.06 ± 0.92	3.61 ± 0.73
	Stress phase	5.65 ± 1.42	5.47 ± 1.20	7.18 ± 0.63	6.19 ± 0.93	3.69 ± 0.72
	Recovery phase	3.73 ± 1.57	5.45 ± 1.17	6.58 ± 0.74	6.08 ± 0.87	3.62 ± 0.71
HTA (*n* = 48)	Baseline phase	3.83 ± 1.55	5.40 ± 1.14	6.29 ± 0.75	6.37 ± 0.83	3.77 ± 0.95
	Stress phase	6.73 ± 1.32	4.27 ± 1.62	6.58 ± 0.73	5.79 ± 0.91	3.58 ± 1.09
	Recovery phase	4.33 ± 1.36	5.06 ± 1.08	6.40 ± 0.60	6.34 ± 0.78	3.80 ± 0.95

**FIGURE 2:**
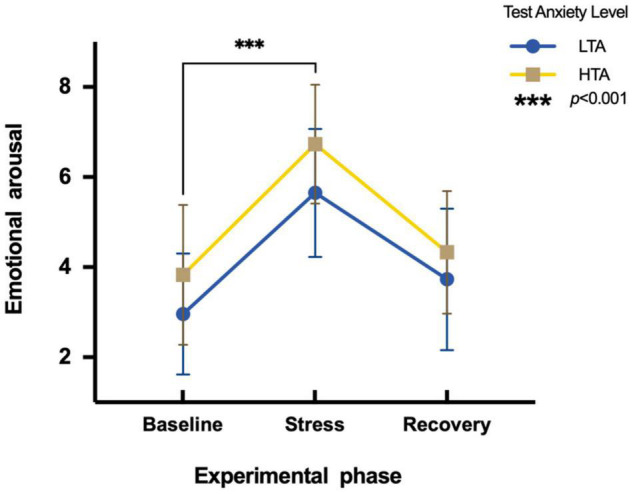
Differences in emotional arousal during three experimental phases of HTA and LTA individuals. *** represents a significant difference between the baseline and stress phase of both groups with *p*-value < 0.001.

To assess the differences in emotional arousal between HTA and LTA individuals during three experimental phases, a two-factor repeated measures ANOVA is conducted. Results show a main significant effect in the experimental phase [*F*(2,95) = 157.59, *p* < 0.001, η*^2^_*p*_* = 0.62]. Subjects show significantly higher emotional arousal during the stress phase (*M* = 6.19, *SE* = 1.47) than during the baseline phase (*M* = 3.39, *SE* = 1.50). Additionally, there is a significant main effect in test anxiety [*F*(1,95) = 15.09, *p* < 0.001, η*^2^_*p*_* = 0.14]. The emotional arousal of HTA individuals (*M* = 4.97, *SE* = 1.89) is significantly higher than that of LTA subjects (*M* = 4.12, *SE* = 1.83). The interaction is not significant [*F*(2,95) = 1.06, *p* > 0.05, η*^2^_*p*_* = 0.01] (Figure 2).

**FIGURE 3:**
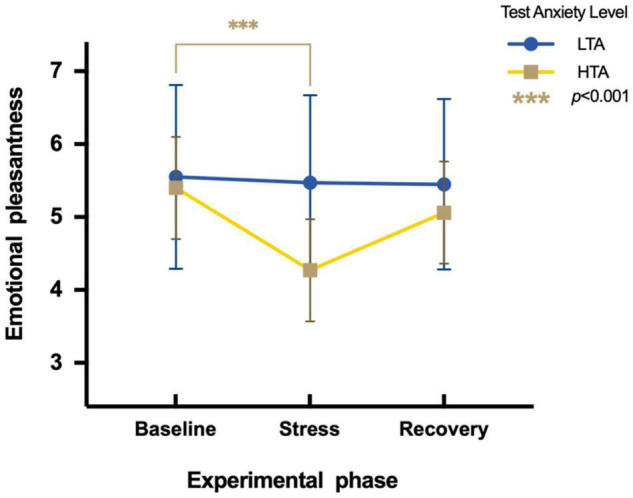
Differences in emotional pleasantness during three experimental phases of HTA and LTA individuals. *** represents a significant difference between the baseline and stress phase of HTA individuals with *p*-value < 0.001.

To assess differences in emotional pleasantness between HTA and LTA individuals during three experimental phases, a two-factor repeated measures ANOVA is conducted. Results show a significant main effect in the experimental phase [*F*(2,95) = 8.19, *p* < 0.01, η*^2^_*p*_* = 0.08] and test anxiety levels [*F*(1,95) = 7.33, *p* < 0.01, η*^2^_*p*_* = 0.07]. The interaction is significant [*F*(2,95) = 5.63, *p* < 0.05, η*^2^_*p*_* = 0.06]. Simple effect analysis indicates that LTA subjects show no significant differences in emotional pleasantness during three experimental phases [*F*(2,48) = 0.19, *p* > 0.05, η*^2^_*p*_* = 0.004]. HTA subjects show significant differences in emotional pleasantness during three experimental phases [*F*(2,47) = 12.92, *p* < 0.001, η*^2^_*p*_* = 0.22]. Their emotional pleasantness during the stress phase (*M* = 4.27, *SE* = 1.62) is significantly lower than during the baseline phase [*M* = 5.40, *SE* = 1.14; *t* (47) = −4.26, *p* < 0.001, Cohen’*d* = −0.81] (Figure 3).

**FIGURE 4:**
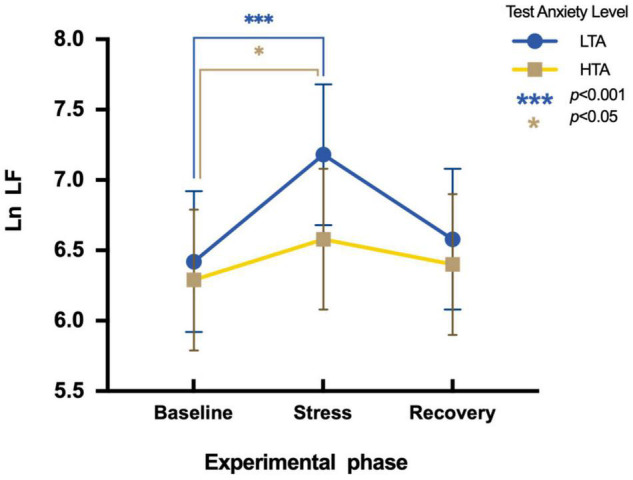
Differences in Ln LF during three experimental phases of HTA and LTA individuals. *** represents a significant difference between the baseline and stress phase of LTA individuals with *p*-value < 0.001. * represents a significant difference between the baseline and stress phase of HTA individuals with *p*-value < 0.05.

To assess differences in Ln LF between HTA and LTA individuals during three experimental phases, a two-factor repeated measures ANOVA is conducted. Results show a main significant effect in the experimental phase [*F*(2,95) = 27.01, *p* < 0.001, η*^2^_*p*_* = 0.22] and in the test anxiety level [*F*(1,95) = 6.77, *p* < 0.05, η*^2^_*p*_* = 0.07]. The interaction is significant [*F*(2,95) = 5.85, *p* < 0.001, η*^2^_*p*_* = 0.06]. Simple effect analysis indicates that LTA subjects significantly vary in Ln LF during three experimental phases [*F*(2,48) = 42.84, *p* < 0.001, η^2^_*p*_ = 0.48]. Their Ln LF values during the stress phase (*M* = 7.18, *SE* = 0.03) are significantly higher than that during the baseline phase [*M* = 6.42, *SE* = 0.83; *t* (48) = 7.55, *p* < 0.001, Cohen’*d* = 0.57]. Nevertheless, there is a marginally significant difference in Ln LF values of HTA subjects during different experimental phases [*F*(2,47) = 3.01, *p* = 0.06, η*^2^_*p*_* = 0.22]. Their Ln LF values during the stress phase (*M* = 6.58, *SE* = 0.73) are significantly higher than during the baseline phase [*M* = 6.29, *SE* = 0.75; *t* (47) = 2.01, *p* < 0.05, Cohen’*d* = 0.39] (Figure 4).

**FIGURE 5:**
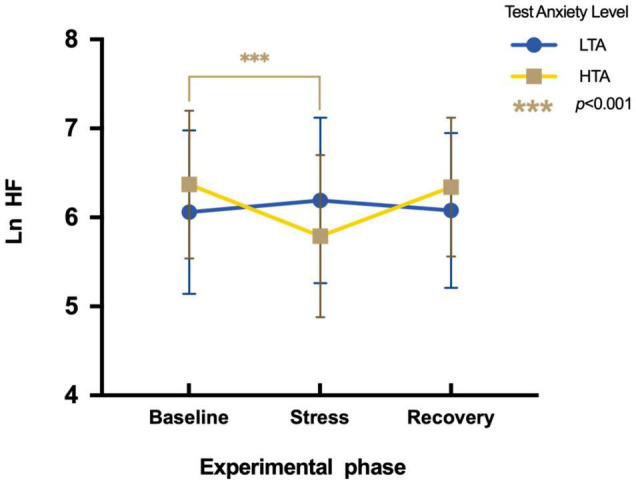
Differences in Ln HF during three experimental phases of HTA and LTA individuals. *** represents a significant difference between the baseline and stress phase of HTA individuals with *p*-value < 0.001.

To assess differences in Ln HF between HTA and LTA individuals during three experimental phases, a two-factor repeated measures ANOVA is conducted. Results show a significant main effect in the experimental phase [*F*(2,95) = 8.54, *p* < 0.01, η*^2^_*p*_* = 0.08], whereas there is no significant main effect in the test anxiety level [*F*(1,95) = 0.11, *p* > 0.05, η^2^_*p*_ = 0.001]. The interaction is significant [*F*(2,96) = 20.21, *p* < 0.001, η*^2^_*p*_* = 0.18]. Simple effect analysis shows that LTA subjects display no significant difference in Ln HF during three experimental phases [*F*(2,48) = 1.71, *p* > 0.05, η*^2^_*p*_* = 0.03], whereas HTA subjects vary significantly during three experimental phases [*F*(2,47) = 21.56, *p* < 0.01, η*^2^_*p*_* = 0.31]. Their Ln HF values during the stress phase (*M* = 5.79, *SE* = 0.91) are significantly lower than during the baseline phase [*M* = 6.37, *SE* = 0.83; *t* (47) = −5.48, *p* < 0.001, Cohen’*d* = −0.67] (Figure 5).

**FIGURE 6:**
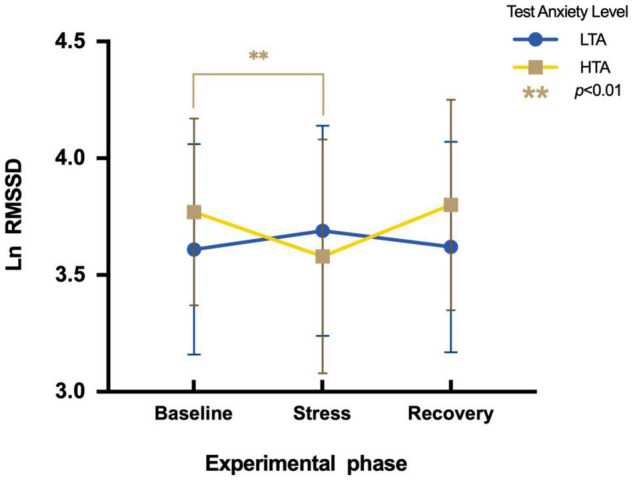
Differences in Ln RMSSD during three experimental phases of HTA and LTA individuals. ** represents a significant difference between the baseline and stress phase of HTA individuals with *p*-value < 0.01.

To assess differences in Ln RMSSD between HTA and LTA individuals during three experimental phases, a two-factor repeated measures ANOVA is conducted. There is no significant main effect in the experimental stage [*F*(2,95) = 2.39, *p* > 0.05, η*^2^_*p*_* = 0.03] and in the test anxiety level [*F*(1,95) = 0.66, *p* > 0.05, η*^2^_*p*_* = 0.002]. The interaction is significant [*F*(2,95) = 9.70, *p* < 0.001, η*^2^_*p*_* = 0.09]. Simple effect analysis indicates that LTA subjects show no significant differences in Ln RMSSD during three experimental phases [*F*(2,48) = 1.66, *p* > 0.05, η*^2^_*p*_* = 0.03], while HTA subjects vary significantly during three experimental phases [*F*(2,47) = 9.05, *p* < 0.01, η^2^_*p*_ = 0.16]. Their Ln RMSSD values during the stress phase (*M* = 3.58, *SE* = 1.09) are significantly lower than during the baseline phase [*M* = 3.77, *SE* = 0.95; *t* (47) = −3.09, *p* < 0.01, Cohen’*d* = −0.19] (Figure 6).

## Discussion

In this study, we used a standard interview task to induce evaluative stress to explore the ANS response patterns and emotional experience of test-anxious individuals. Results show that emotional arousal and Ln LF values of both groups increase significantly in response to evaluative stress. HTA individuals show a significant decrease in emotional pleasantness, Ln HF, and Ln RMSSD values during the stress phase. At the same time, LTA individuals show no significant changes in these measures during the stress phase.

When both groups are exposed to the evaluative scenario, they have similar emotional experience and SNS responses. We find that the emotional arousal and Ln LF values of both groups are significantly higher during the stress phase than during the baseline phase. These results indicate that the evaluative stressors we set in the experiment could effectively induce stress responses, which is consistent with previous research (Loft et al., 2007; Zhang et al., 2015). More importantly, the increase in Ln LF values indicates increased SNS activity in response to evaluative stress. Stress responses require energy mobilization, a metabolic function served by the SNS, and this response is controlled by sympathetic-mediated changes in the contractile force of the left ventricle (Richter et al., 2012). Increased SNS activity also contributes to the rapid mobilization of dopamine, adrenaline, and norepinephrine secretion (signaling physiological arousals such as accelerated heartbeats and elevated blood pressure) (Strohmaier et al., 2020).

Though HTA and LTA individuals have similarities in emotional experience and the ANS responses, several differences exist in other aspects, which play an important role in the differences between HTA and LTA individuals in the evaluative scenario. From the perspective of emotional experience, the results indicate that only HTA individuals reduce their emotional pleasantness, while LTA individuals remain stable in the evaluative scenario. This result is consistent with previous studies indicating that HTA individuals are more prone to tension in evaluative scenarios and magnify negative emotional experience (Conley and Lehman, 2012; Zeidner, 2014). Negative emotions would occupy considerable cognitive resources according to the Attentional Control Theory, consequently reducing available working memory resources during cognitive processes and thus impairing cognitive processing efficiency (Wei et al., 2021). HTA individuals’ reduced emotional pleasantness can also be interpreted as avoidance behavior, which would decrease available resources to cope with environmental challenges (Zhang et al., 2018; Wei et al., 2021).

From the perspective of the ANS responses, the results indexed by Ln LF and Ln RMSSD indicate that HTA individuals display decreased PNS activity, whereas LTA individuals display stable PNS activity. According to the neurovisceral integration model and the Poly-Vagal theory, PNS activity is regarded as an inhibition underpinned by vagus nerves. The PNS, which serves as floodgates, can regulate heart responses and control individual emotional and social responses (Porges, 2009; Thayer, 2009). Therefore, decreased PNS activity shows the lack of inhibition of HTA individuals in the evaluative scenario (John et al., 2013). To be specific, lack of inhibition means a failure to inhibit cognitive (e.g., vigilance and worry), affective (e.g., panic), behavioral (e.g., avoidance), and physiological (e.g., increased HR) responses, thus resulting in poor performance of HTA individuals in the exam. Compared to the decreased PNS responses of HTA individuals, stable PNS activity indicates that LTA individuals could effectively control the hyperactive responses of sympathetic nerves and maintain a great cognitive, emotional, and behavioral state, conducive to performing their cognitive abilities in an evaluative scenario.

Furthermore, the balance of SNS and PNS contributes to understanding the underlying neural mechanism of test anxiety (John et al., 2013). HTA individuals display increased SNS activity and decreased PNS activity in response to evaluative stress, indicating the inability of the ANS to maintain balance. The imbalance would cause a weakening efficiency in regulating cognitive, emotional, and behavioral functions, thus failing to effectively address the challenge of evaluative stress (Porges, 2009; Thayer, 2009; Wei et al., 2021). Correspondingly, despite the increased SNS activity in the evaluative scenario, the PNS activity of LTA individuals remained stable: this helps maintain the balance of ANS function and thus effectively cope with the environmental challenges.

In this study, HRV measures were used to reflect the ANS’s response patterns of test-anxious individuals, with the aim to gain a better understanding of the neural mechanism of test anxiety. HTA individuals have a more negative emotional experience and a poorer balance of ANS activity (i.e., hyper SNS activity and weakened PNS activity) when confronted with evaluative stress. In the future, in order to alleviate test anxiety problems, biological feedback training can be used to increase the level of PNS activity and improve the balance of ANS activity. In this way, the adverse effects of test anxiety on students’ psychosomatic health and academic performance might be alleviated (Goessl et al., 2017).

## Limitations

Though the findings from the current study are promising, several limitations of the present research should still be considered when interpreting its findings and providing innovative directions for future research. Firstly, our sample is primarily composed of college students, which restricts generalization to other age groups. Future studies can take this limitation into account. Secondly, the experimental scenario may limit the applicability to more naturalistic conditions. Future research should be conducted in a more natural setting to ensure applicability to real-life scenarios.

## Conclusion

This study has shown that test-anxious individuals differ in their ANS response patterns when confronted with evaluative stress. HTA individuals show increased SNS activity and decreased PNS activity, whereas LTA individuals show increased SNS activity and stable PNS activity. By capturing the complex construct of text anxiety, we might profoundly understand the neural mechanism underlying test anxiety. This more ecologically valid, objective, and detailed assessment might also help improve interventions targeting test anxiety.

## Data Availability Statement

The raw data supporting the conclusions of this article will be made available by the authors, without undue reservation.

## Ethics Statement

The study involving human participants was reviewed and approved by the Ethics Committee of the Nanjing University of Chinese Medicine. The participants provided their written informed consent to participate in this study.

## Author Contributions

WB and XZ contributed to the conception and design of the study, and conducted the experiment. YD checked and corrected the scientific issue of the study. WB wrote the first draft of the manuscript and analyzed the data. All authors contributed to the revision of the manuscript and approved it for publication.

## Conflict of Interest

The authors declare that the research was conducted in the absence of any commercial or financial relationships that could be construed as a potential conflict of interest.

## Publisher’s Note

All claims expressed in this article are solely those of the authors and do not necessarily represent those of their affiliated organizations, or those of the publisher, the editors and the reviewers. Any product that may be evaluated in this article, or claim that may be made by its manufacturer, is not guaranteed or endorsed by the publisher.

## References

[B1] Boeke E. A. Moscarello J. M. LeDoux J. E. Phelps E. A. Hartley C. A. (2017). Active Avoidance: neural Mechanisms and Attenuation of Pavlovian Conditioned Responding. *J. Neurosci.* 37 4808–4818. 10.1523/JNEUROSCI.3261-16.2017 28408411 PMC5426570

[B2] Conley K. M. Lehman B. J. (2012). Test anxiety and cardiovascular responses to daily academic stressors: test anxiety and cardiovascular responses. *Stress Health* 28, 41–50. 10.1002/smi.1399 22259157

[B3] Fanselow M. S. (1994). Neural organization of the defensive behavior system responsible for fear. *Psychon. Bull. Rev.* 1 429–438. 10.3758/BF03210947 24203551

[B4] Goessl V. C. Curtiss J. E. Hofmann S. G. (2017). The effect of heart rate variability biofeedback training on stress and anxiety: a meta-analysis. Psychol. Med. 47, 2578-2586. 10.1017/S0033291717001003 28478782

[B5] Hamm A. O. Weike A. I. (2005). The neuropsychology of fear learning and fear regulation. *Int. J. Psychophysiol.* 57 5–14. 10.1016/j.ijpsycho.2005.01.006 15935258

[B6] Heiss S. Vaschillo B. Vaschillo E. G. Timko C. A. Hormes J. M. (2021). Heart rate variability as a biobehavioral marker of diverse psychopathologies: a review and argument for an “ideal range.”. *Neurosci. Biobehav. Rev.* 121 144–155. 10.1016/j.neubiorev.2020.12.004 33309905

[B7] Hollandsworth J. G. Glazeski R. C. Kirkland K. Jones G. E. Van Norman L. R. (1979). An analysis of the nature and effects of test anxiety: cognitive, behavioral, and physiological components. *Cogn. Ther. Res.* 3 165–180. 10.1007/BF01172603

[B8] Holzman J. B. Bridgett D. J. (2017). Heart rate variability indices as bio-markers of top-down self-regulatory mechanisms: a meta-analytic review. *Neurosci. Biobehav. Rev.* 74, 233–255. 10.1016/j.neubiorev.2016.12.032 28057463

[B9] Huang Q. Zhou R. L. (2019). The Development of Test Anxiety in Chinese Students. *Chinese J. Clin. Psychol.* 27 113–118. 10.16128/j.cnki.1005-3611.2019.01.023

[B10] Huntley C. D. Young B. Temple J. Longworth M. Smith C. T. Jha V. (2019). The efficacy of interventions for test-anxious university students: a meta-analysis of randomized controlled trials. *J. Anxiety Disord.* 63 36–50. 10.1016/j.janxdis.2019.01.007 30826687

[B11] John C. Daniel Q. Maree A. Andrew K. (2013). “The impact of anxiety and its disorders on heart rate variability: a meta-analysis,” in *Conference Abstract: ASP2013 - 23rd Annual meeting of the Australasian Society for Psychophysiology*, (Australia: University of Sydney). 10.3389/conf.fnhum.2013.213.00008

[B12] Krypotos A.-M. (2015). Avoidance learning: a review of theoretical models and recent developments. *Front. Behav. Neurosci.* 9:189. 10.3389/fnbeh.2015.00189 26257618 PMC4508580

[B13] Lang J. W. B. Lang J. (2010). Priming competence diminishes the link between cognitive test anxiety and test performance: implications for the interpretation of test scores. *Psychol. Sci.* 21, 811–819. 10.1177/0956797610369492 20435953

[B14] Lang P. J. Bradley M. M. Cuthbert B. N. (1997). “Motivated attention: affect, activation, and action,” in *Attention and Orienting: Sensory and Motivational Processes*, eds Lang P. J. Simons R. Balaban M. T. (Mahwah, NJ: Lawrence Erlbaum Associates), 97–136.

[B15] Loft P. Thomas M. G. Petrie K. J. Booth R. J. Miles J. Vedhara K. (2007). Examination stress results in altered cardiovascular responses to acute challenge and lower cortisol. *Psychoneuroendocrinology* 32 367–375. 10.1016/j.psyneuen.2007.02.004 17395393

[B16] Lü W. Xing W. Hughes B. M. Wang Z. (2018). Extraversion and cardiovascular responses to recurrent social stress: effect of stress intensity. *Int. J. Psychophysiol.* 131, 144–151. 10.1016/j.ijpsycho.2017.10.008 29111452

[B17] Porges S. W. (1992). Vagal tone: a physiological marker of stress vulnerability. *Pediatrics* 90 498–504. 10.1542/peds.90.3.498 1513615

[B18] Porges S. W. (2007). The polyvagal perspective. *Biol. Psychol.* 74 116–143. 10.1016/j.biopsycho.2006.06.009 17049418 PMC1868418

[B19] Porges S. W. (2009). The polyvagal theory: new insights into adaptive reactions of the autonomic nervous system. *Cleve. Clin. J. Med.* 76 S86–S90. 10.3949/ccjm.76.s2.17 19376991 PMC3108032

[B20] Putwain D. W. Gallard D. Beaumont J. Loderer K. von der Embse N. P. (2021). Does test anxiety predispose poor school-related wellbeing and enhanced risk of emotional disorders? *Cogn. Ther. Res.* 45, 1150–1162. 10.1007/s10608-021-10211-x

[B21] Ramirez G. Beilock S. L. (2011). Writing about testing worries boosts exam performance in the classroom. *Science* 331, 211–213. 10.1126/science.1199427 21233387

[B22] Richter J. Hamm A. O. Pané-Farré C. A. Gerlach A. L. Gloster A. T. Wittchen H.-U. (2012). Dynamics of Defensive Reactivity in Patients with Panic Disorder and Agoraphobia: Implications for the Etiology of Panic Disorder. *Biol. Psychiatry* 72 512–520. 10.1016/j.biopsych.2012.03.035 22621998

[B23] Roos A.-L. Goetz T. Voracek M. Krannich M. Bieg M. Jarrell A. (2021). Test Anxiety and Physiological Arousal: a Systematic Review and Meta-Analysis. *Educ. Psychol. Rev.* 33 579–618. 10.1007/s10648-020-09543-z

[B24] Spielberger C. D. (1980). *Test Anxiety Inventory:Preliminary Professional Manual.* California, CA: Consulting Psychology Press.

[B25] Strohmaier A. R. Schiepe-Tiska A. Reiss K. M. (2020). A comparison of self-reports and electrodermal activity as indicators of mathematics state anxiety. *Front. Learn Res.* 8 16–32. 10.14786/flr.v8i1.427

[B26] Sutarto A. P. Wahab M. N. A. Zin N. M. (2013). Effect of biofeedback training on operator’s cognitive performance. *Work* 44 231–243. 10.3233/WOR-121499 23324677

[B27] Thayer J. F. (2009). Heart Rate Variability: a Neurovisceral Integration Model. *Encyclopedia Neurosci.* 2009 1041–1047. 10.1016/B978-008045046-9.01991-4

[B28] Wang C. K. (2003). The Reliability and Validation of Chinese Version Test Anxiety Inventory in College Students. *Chin. J. Clin. Psychol.* 11 69–70.

[B29] Wei H. De Beuckelaer A. Zhou R. (2021). Enhanced or impoverished recruitment of top-down attentional control of inhibition in test anxiety. *Biol. Psychol.* 161:108070. 10.1016/j.biopsycho.2021.108070 33722566

[B30] Wei L. (2020). Differential effects of avoidance and approach negative personality traits on patterns of stress cardiovascular responses. *Acta Psychol. Sin.* 52 758–776. 10.3724/SP.J.1041.2020.00758

[B31] Wells R. Outhred T. Heathers J. A. J. Quintana D. S. Kemp A. H. (2012). Matter Over Mind: a Randomised-Controlled Trial of Single-Session Biofeedback Training on Performance Anxiety and Heart Rate Variability in Musicians. *PLoS One* 7:e46597. 10.1371/journal.pone.0046597 23056361 PMC3464298

[B32] Yan K. L. Zhang W. C. Zhang Y. J. Feng W. B. Yuan L. Z. Wang L. S. (2006). The Application of Heart Rate Variability to Study Psychosomatic Disease and Emotion Disorder. *Adv. Psycholo. Sci.* 14 261–265.

[B33] Yoshie M. Kudo K. Murakoshi T. Ohtsuki T. (2009). Music performance anxiety in skilled pianists: effects of social-evaluative performance situation on subjective, autonomic, and electromyographic reactions. *Exp. Brain Res.* 199 117–126. 10.1007/s00221-009-1979-y 19701628

[B34] Zeidner M. (2014). “Introduction to emotions in education,” in *International Handbook of Emotions in Education*, eds Pekrun R. Linnenbrink-Garcia E. A. (New York, NY: Taylor & Francis).

[B35] Zhang X. Dong Y. Zhou R. (2018). Examination Stress Results in Attentional Bias and Altered Neural Reactivity in Test-Anxious Individuals. *Neural. Plast.* 2018:3281040. 10.1155/2018/3281040 29755511 PMC5884033

[B36] Zhang X. C. Zou J. L. Dong Y. Y. Zhang H. Zhou R. L. (2015). Effects of Test Pressure on Working Memory Capacity in College Students with High Test Anxiety. *Chin. J. Clin. Psychol.* 23 635–638. 10.16128/j.cnki.1005-3611.2015.04.015

